# Macrophages induce gingival destruction via Piezo1-mediated MMPs-degrading collagens in periodontitis

**DOI:** 10.3389/fimmu.2023.1194662

**Published:** 2023-05-16

**Authors:** Tong Zhao, Zhuangzhuang Chu, Catherine Huihan Chu, Shuo Dong, Guoqing Li, Jin Wu, Chunbo Tang

**Affiliations:** ^1^ Department of Dental Implantology and Prosthodontics, Affiliated Hospital of Stomatology, Nanjing Medical University, Nanjing, Jiangsu, China; ^2^ Key Laboratory of Oral Diseases, Jiangsu Province Engineering Research Center of Stomatological Translational Medicine, Nanjing, China; ^3^ Department of Dental Orthodontic, Affiliated Hospital of Stomatology, Nanjing Medical University, Nanjing, Jiangsu, China

**Keywords:** periodontitis, Piezo1, gingival destruction, macrophage, matrix metalloproteinases

## Abstract

Macrophages are an integral part of the innate immune response in periodontal tissue and play a crucial role in the progression of periodontitis. Here we reported that macrophages also provoke periodontitis-induced gingival destruction through Piezol-mediated collagen degradation. We discovered that the *PIEZO1* expression was markedly elevated in patients with periodontitis through transcriptomic profiling. Moreover, Piezo1 promoted macrophage polarization toward the M1 type in response to lipopolysaccharide (LPS) and induced production of proinflammatory cytokines, which in turn stimulated production of matrix metalloproteinases (MMPs) leading to collagen degradation. Our study suggests that Piezol might be a potential therapeutic target for treating periodontitis-induced gingival destruction.

## 1 Introduction

Periodontitis is a common infectious periodontal disease caused by plaque biofilm, which affects millions of individuals every year and ranks as the sixth most prevalent disease in the world (1). An epidemiological survey reveals that there are hundreds of millions of patients with periodontitis in China, of which 20 - 30% have advanced to the stage of severe periodontitis (2). It is suggested that microbe and their products (e. g. LPS) in periodontal tissues, which include gingiva, periodontal ligament, alveolar bone and cementum, activate innate immunity (3, 4) and trigger periodontitis and subsequent irreversible damage, periodontal attachment loss, gingival atrophy, alveolar bone resorption, and teeth loosening and loss (5). However, the underlying molecular mechanisms are still not well understood.

Macrophages are key components of innate immunity (6). While macrophages accumulate at the site of microbial infection, recognize, and phagocytose microbial pathogens, contributing to the homeostasis of the tissue microenvironment, they also trigger abnormal immune responses to microbe in the gingival tissue causing collagen fibers to deteriorate and consequent gingival atrophy (7, 8). However, how macrophages stimulate the pathogenic process is unclear.

Piezo type mechanosensitive ion Channel component 1 (Piezol) is a recently identified mechanosensitive ion channel that converts mechanical signals into biochemical responses (9, 10). A variety of organs, tissues and cells in the human body can sense mechanical stimuli in the external environment and activate cellular signal transduction pathways through Piezo1 (9, 11). Piezo1 is associated with orthodontic tooth movement to enhance alveolar bone remodeling (12, 13). Studies have also demonstrated an inextricable link between Piezo1 and inflammatory responses (14) (15). Piezo1 is also a mechanosensor of stiffness of macrophages and regulates macrophage function (16)). However, the role of Piezo1 in macrophages-mediated periodontal inflammation remains elusive.

In this study, we used RNA sequencing (RNA-seq) and other approaches (e. g., molecular biology techniques, immunofluorescence techniques) to analyze gene expression profiles of gingival tissues from periodontitis patients and healthy individuals, revealing new insights into the mechanisms underlying macrophage and periodontal tissue destruction in periodontitis.

## 2 Methods and materials

### 2.1 Study subjects and tissue samples

A total of 14 subjects participated in this study, including 7 patients with periodontitis and 7 healthy individuals. All subjects were enrolled from the Department of Oral and Maxillofacial Surgery, Nanjing Medical University Oral Hospital. The inclusion criteria for periodontitis group were as follows (1): probing depth (PD) of ≥4mm (2); clinical attachment level (CAL) of ≥5mm (at site of greatest loss) (3); gingival recession of grade I and above according to Miller’s classification (4); bleeding on probing (BOP) in the area affected by periodontitis (5); radiographic examination showed loss of alveolar bone. Healthy controls were included for those who required gingival flap surgery to extract their impacted wisdom teeth. All subjects had no history of systemic diseases and inflammatory disorders such as hypertension, diabetes, cerebrovascular disease (CVD), inflammatory bowel disease (IBD), etc. Besides, all subjects were not pregnant or breastfeeding and had no history of smoking. None of the subjects had taken drugs such as antibiotics or immunosuppressants in the previous 3 months. The gingival samples of the periodontitis group were collected from the areas of gingival recession. All gingival samples collected were approximately 0. 5 cm^2^ in size and included epithelial and connective tissue. Following rapid washing with phosphate buffer to remove blood, the tissues were immediately transferred into dry 2 mL Eppendorf tubes, temporarily stored in liquid nitrogen, and later stored at -80°C until subsequent procedures. This study was approved by the Medical Ethics Committee of the School of Stomatology, Nanjing Medical University and the Affiliated Stomatology Hospital of Nanjing Medical University with approval number PJ2021-126-001, and informed consent was acquired from each patient prior to the procedure.

### 2.2 RNA sequencing and sequencing data analysis

Total RNA was extracted from the gingival tissue employing TRIzol reagent (Invitrogen, USA) following the manufacturer’s protocol after accurately weighing 30 mg of each sample. Then the RNA purity was determined by NanoDrop 2000 spectrophotometer (Thermo Fisher Scientific, USA), and the RNA integrity was evaluated by Agilent 2100 Bioanalyzer (Agilent Technologies, USA). The TruSeq Stranded mRNA LTSample Prep Kit (Illumina, USA) was used to undertake cDNA synthesis and library creation according to the manufacturer’s protocol. The constructed libraries were quality-checked with Agilent 2100 Bioanalyzer and sequenced by the Illumina HiSeq6000 sequencer after passing quality check. Trimmomatic software was used to quality control the raw data (Raw Reads) to obtain Clean Reads. The acquired Clean Reads were compared with the human genome GRCh38 for sequence comparison by HISAT2 to obtain sample-specific sequence feature information. The gene expression was calculated by the Fragments Per kb Per Million Reads (FPKM) method. Then the differentially expressed genes (DEGs) between periodontitis group and healthy group were identified by the limma package in R software with the threshold of |log_2_FC (fold**-**change)| > 1 and P < 0. 05. GO enrichment and KEGG pathway enrichment analyses were conducted on DEGs, and the significance of DEGs enrichment was determined by the hypergeometric test. Sequencing and analysis of the transcriptome were performed by OE Biotech Co. (Shanghai, China).

### 2.3 Cell culture and treatment

RAW264. 7, a mouse-derived mononuclear macrophage leukemia cell line (Cell Bank, Chinese Academy of Sciences, Shanghai, China), was seeded in cell culture flasks and cultured in high-glucose Dulbecco’s modified Eagle’s medium (DMEM) (Gibco, USA) containing 1% penicillin and streptomycin (Gibco, USA) and 10% fetal bovine serum (FBS) (Gibco, USA), maintained at 37°C in a humidified atmosphere with 5% CO_2_, and passaged every 2 days at a ratio of 1:2.

Human gingival fibroblasts (HGF-1) were purchased from Shanghai Honsun Biological Technology Co., Ltd (Shanghai, China). The culture conditions for HGF-1 cells were the same as those for RAW264. 7 cells. HGF-1 cells were passaged every 3 days at a ratio of 1:3.

**Figure 1:**
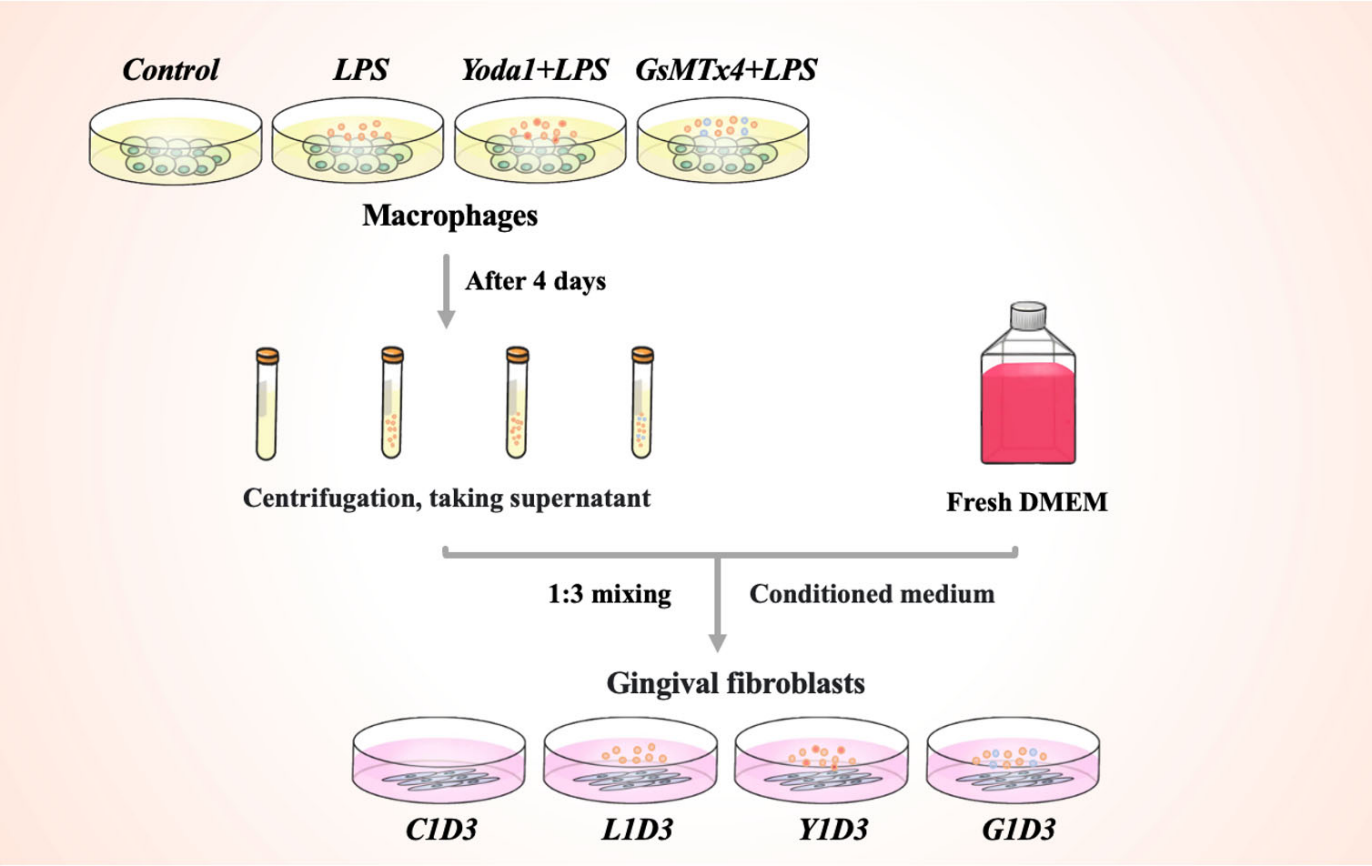
Schematic diagram of the conditional culture process.

RAW264. 7 cells were seeded at a density of 1×10^5^ in 6-well plates for 4 days. Porphyromonas gingivalis (P. g) is the main causative agent of periodontitis (17). P. g-LPS (100 ng/mL, SMB00610, Sigma-Aldrich, USA) induced macrophages to stimulate the periodontal inflammatory environment. Yoda1 and GsMTx4 were purchased from MedChemExpress (HY-18723, USA) and AbMole (M10039, USA). In the presence of LPS, Yoda1 (5μM) or GsMTx4 (4μM) were added to RAW264. 7 cells to induce or inhibit Piezo1 expressions. Negative controls were RAW264. 7 cell culture medium. Groups of cells were cultured for up to 4 days without changing the culture medium during this period. The groups of RAW264. 7 cells were named Control, LPS, Yoda1+LPS and GsMTx4+LPS, respectively. Then each group’s medium was collected, and the supernatant rich in macrophage-derived cytokines was obtained after centrifugation. The aforementioned macrophage supernatant was mixed with fresh DMEM medium at a ratio of 1:3 to prepare a conditioned medium for the cultivation of HGF-1 cells to confirm that macrophages would immunomodulate gingival tissue. The groups of HGF-1 cells were named C1D3, L1D3, Y1D3 and G1D3, respectively. The schematic diagram of HGF-1 cell cultured with macrophage conditioned medium was shown in **Figure 1**.

### 2.4 Cell viability assay

A cell counting kit-8 assay (CCK8, Dojindo, Japan) was performed to determine the viability of RAW264. 7 cultured with reagents and HGF-1 cultured with conditioned medium. Cells were seeded onto 24-well plates at an initial density of 5×10^4^ cells per well. At each assay time point, the medium was replaced with fresh medium containing 10% CCK-8 solution and the cells were incubated for 2 hours at 37°C in the absence of light. The absorbance optical density (OD) values were then measured with a microplate reader at the wavelength of 450nm.

### 2.5 Immunofluorescence (IF) staining

#### 2.5.1 Tissue staining

Fresh gingival tissue was briefly submerged in PBS to remove residual blood before being preserved in 4% paraformaldehyde overnight at 4°C. The tissues were then washed three times in PBS for 30 minutes each before being embedded in paraffin. Paraffin slices were dewaxed in an environmentally friendly dewaxing solution and subsequently hydrated with anhydrous ethanol and distilled water. Tissue sections were placed in a repair cassette filled with EDTA antigen repair buffer (pH=8. 0) in a microwave oven for antigen repair. The sections were blocked with goat serum for 30 minutes and then incubated overnight with Piezo1 antibody (1:100; NBP1-78446; Novus Biologicals, USA) and CD68 primary antibody (1:50; ab955; Abcam, UK) at 4°C in a wet box. After immersing sections in PBS (pH=7. 4) on a decolorization shaker and rinsing 3 times for 5 minutes each time, the donkey anti-mouse secondary antibody IgG H&L Alexa Fluor 594 (1:200; ab150108; Abcam, UK) and the goat anti-rabbit IgG H&L Alexa Fluor 488 (1:200; ab150077; Abcam, UK) secondary antibodies were added to cover the tissue and incubated for one hour at room temperature under protection from light. Next, add 4’,6-diamidino-2-phenylindole (DAPI) (ab228549; Abcam, USA) to stain the nuclei and incubate for 10 minutes at room temperature in the dark. Finally, the slices were sealed with an anti-fluorescence quenching sealer and imaged under a confocal laser scanning microscope (Leica Microsystems, Germany). Image J software was used to analyze the fluorescence data quantitatively.

#### 2.5.2 Cell staining

RAW264. 7 or HGF-1 cells seeded in confocal dishes were fixed with 4% paraformaldehyde at 4°C for 15 minutes after washed by PBS 3 times for 20 minutes each time. The cells were then permeabilized with 0. 2% Triton X-100 for 10 minutes and blocked with 2% BSA for 2 hours, and each step was followed by twice PBS washes to remove residual reagents. After the above steps were completed, primary antibodies were added to the cells and incubated at 4°C overnight. The primary antibodies were added as follows: Piezo1 antibody and CD68 were co-incubated to localize Piezo1 on macrophages; CCR7 (1:200;NB100-712; Novus Biologicals, USA) and CD206 (1:200; #24595; Cell Signaling Technology, USA) were co-incubated to identify macrophage polarization; COL-I (1:200;NB600-408; Novus Biologicals, USA) or COL-III (1:200; NB600-594; Novus Biologicals, USA) was incubated to indicate the existence of collagen in HGF-1. Afterwards, the cells were incubated with donkey anti-goat secondary antibody IgG H&L Alexa Fluor 594 (1:200; ab150132; Abcam, UK) and the goat anti-rabbit IgG H&L Alexa Fluor 488 for one hour at room temperature in the dark. After washing away the secondary antibodies with PBS, DAPI was used to stain the nuclei for 10 minutes in the absence of light. Finally, the images were acquired by the confocal laser scanning microscope and quantitatively analyzed by Image J software.

### 2.6 Flow cytometry

Flow cytometry was used for the analysis of M1/M2 macrophage. The M1 macrophages were labeled with F4/80^+^/iNOS^+^ and the M2 macrophages were labeled with F4/80^+^/CD206^+^. RAW264. 7 cells of each group were seeded with a density of 1×10^5^ cells per well in a 6-well plate and incubated at 37°C for 4 days. After collected and resuspended with PBS containing 5% BSA, the cells were incubated with phycoerythrin (PE)-conjugated F4/80 monoclonal antibody (2142861,eBiosience™, USA), allophycocyanin (APC)-conjugated CD206 monoclonal antibody (2324767, eBiosience, USA) and Alexa Fluor 488-conjugeted iNOS monoclonal antibody (2365862, eBiosience, USA). The samples were then tested by a FACScan flow cytometer (Becton Dickinson, USA). The data was analyzed using FlowJo software.

### 2.7 RNA extraction and quantitative real-time polymerase chain reaction (qRT-PCR) analysis

RAW264. 7 cells of each group were seeded with a density of 5×10^4^ cells per well in a 24-well plate and incubated at 37°C for 4 days. HGF-1 cells of each group were seeded with a density of 5×10^4^ cells per well in a 24-well plate and incubated at 37°C for 7 days. Total RNA was extracted from cells using the TaKaRa MiniBEST Universal RNA Extraction KIT (TaKaRa, Japan) following the manufacturer’s protocol. An absorbance ratio of 260:280 was used to evaluate RNA purity, and its concentration was calculated by the absorbance at 260 nm. Total RNA was immediately reverse-transcribed into first-strand cDNA by the TaKaRa PrimeScript™RT Master Mix (TaKaRa, Japan). The obtained cDNA was stored at -20°C and used as soon as possible. As a qRT-PCR reaction template, the cDNA was used SYBR Premix Ex Taq II (TaKaRa, Japan) to amplify target genes on a Q7 system (QuantStudio 7, Applied Biosystems, USA). The reaction was carried out in a program of 95°C for 10 minutes and 40 cycles of 95°C for 15 seconds and 60°C for 1 minute and 95°C for 15 seconds, 60°C for 1 minute. 2^−ΔΔCt^ analysis method was used for calculating the relative expression of target genes. The target genes expressions were normalized to housekeeping gene β-actin. The primer sequences for qRT-PCR analysis were purchased from BioTNT (Shanghai, China) and were shown in **Tables S1, S2**.

### 2.8 Protein extraction and western blotting analysis

RAW264. 7 cells of each group were seeded with a density of 1×10^5^ cells per well in a 6-well plate and incubated at 37°C for 4 days. To extract total protein, cells were washed twice with PBS and then lysed in RIPA buffer (Beyotime Biotechnology, China) supplemented with 1mM phenylmethylsulfonyl fluoride (PMSF) (Beyotime Biotechnology, China) on ice. Quantification of protein concentration was then performed by bicinchoninic acid (BCA) protein assay (Beyotime Biotechnology, China). Protein samples were loaded onto 10% sodium dodecyl sulfate-polyacrylamide (SDS-PAGE) gel with equal amounts per lane for electrophoresis and then transferred onto 0. 22μm polyvinylidene difluoride (PVDF) membranes (Millipore, USA) with a semi‐dry transfer apparatus (BioRad, USA). The membranes were immersed in Tris buffered saline-Tween 20 (TBST) with 5% dehydrated milk, dissolved for 2 hours and then incubated with primary antibodies overnight at 4°C on a transference seesaw shaker. Primary antibodies details are as follows: Piezo1 antibody (NBP1-78446), Anti-TNF-α antibody (ab183218, Abcam, USA), Anti-IL-1β antibody (ab216995, Abcam, USA), Anti-β-actin (ab8226,Abcam, USA). The internal control was β-actin. Afterwards, the membranes were washed with TBST buffer thrice to remove primary antibodies and incubated with horseradish peroxidase-conjugated anti-rabbit or anti-mouse IgG (Promega, USA) for one hour. After washed in TBST buffer, ECL detection reagents (Thermo Fisher Scientific, USA) were used to visualize the blots, and Image J software was used to analyze the results.

### 2.9 Intracellular reactive oxygen species (ROS) assay

ROS in macrophage was determined by a ROS Assay Kit (Beyotime Biotechnology, China) according to the manufacturer’s protocol. RAW264. 7 cells of each group were seeded with a density of 5×10^4^ cells per well in a 24-well plate and incubated at 37°C for 4 days. After two washes with PBS, 2′,7′-dichlorodihydrofluorescein diacetate (DCFH-DA) diluted with serum-free culture medium was applied to the cell surface and incubated at 37°C for 20 minutes. An excitation wavelength of 480nm and an emission wavelength of 525nm were chosen to detect fluorescence intensity with a fluorescent microplate reader (SpectraMaxM2e, Molecular Devices, USA). The test was performed on day 1 and day 4 of RAW264. 7 culture.

### 2.10 Enzyme-linked immunosorbent assay (ELISA)

RAW264. 7 cells of each group were seeded with a density of 5×10^4^ cells per well in a 24-well plate and incubated at 37°C for 4 days. Macrophage culture solution was gathered in accordance with 2. 3 and utilized to quantify the concentrations of macrophage-derived pro-inflammatory cytokines TNF-α and IL-1β by the ELISA kits (D721217, D721017; Sangon Biotech, China) following the manufacturer’s protocol. To detect the level of MMPs secreted by HGF-1, HGF-1 cells of each group were seeded with a density of 5×10^4^ cells per well in a 24-well plate and incubated at 37°C for 7 days. After collecting cell cultures, the levels of MMP8 and MMP13 were determined using the ELISA assay kits (ELK1172,ELK2185; ELK Biotechnology, China) following the manufacturer’s protocol. Standard curves were used to calculate cytokines concentrations. The absorbance OD values were then measured with a microplate reader at the wavelength of 450nm. The standard curve was plotted using ELISACalc software, with the concentration of the standard as the horizontal coordinate and the absorbance OD value as the vertical coordinate.

### 2.11 Statistical analysis

All data in this study was expressed as mean ± standard deviation (SD). Prism 9. 4 software (GraphPad Software, USA) was employed to conduct the statistical analysis. An analysis of statistical significance was conducted using the Student’s *t*-test with a value of P < 0. 05 being considered statistically significant.

## 3 Results

### 3.1 Clinical characteristics

This study included 14 individuals, including 7 patients with severe periodontitis and 7 healthy periodontal controls. The clinical characteristics of the patients in this study were summarized in **Table 1**. The mean age of periodontitis group and control group was 46. 7 and 39. 6 years, respectively. Patients in the periodontitis group were slightly older than the healthy group, but the differences were not statistically significant (P > 0. 05). The mean PD of periodontitis group was 5. 57 mm, including a sample with a 7-mm PD (**Table S3**).

**Table 1:** Clinical characteristics of the study population.

Group	Periodontitis (n=7)	Control (n=7)
**Age (years)**	46. 7 ± 5. 5	39. 6 ± 3. 7
**Females (%)**	42. 86	57. 14
**PD (mm)**	5. 57 ± 0. 84	2. 43 ± 0. 45
**CAL (mm)**	7. 14 ± 1. 21	0
**BOP sites**	26%	2%

### 3.2 RNA sequencing analysis of gene expressions in normal and periodontitis gingival tissues

**Figure 2:**
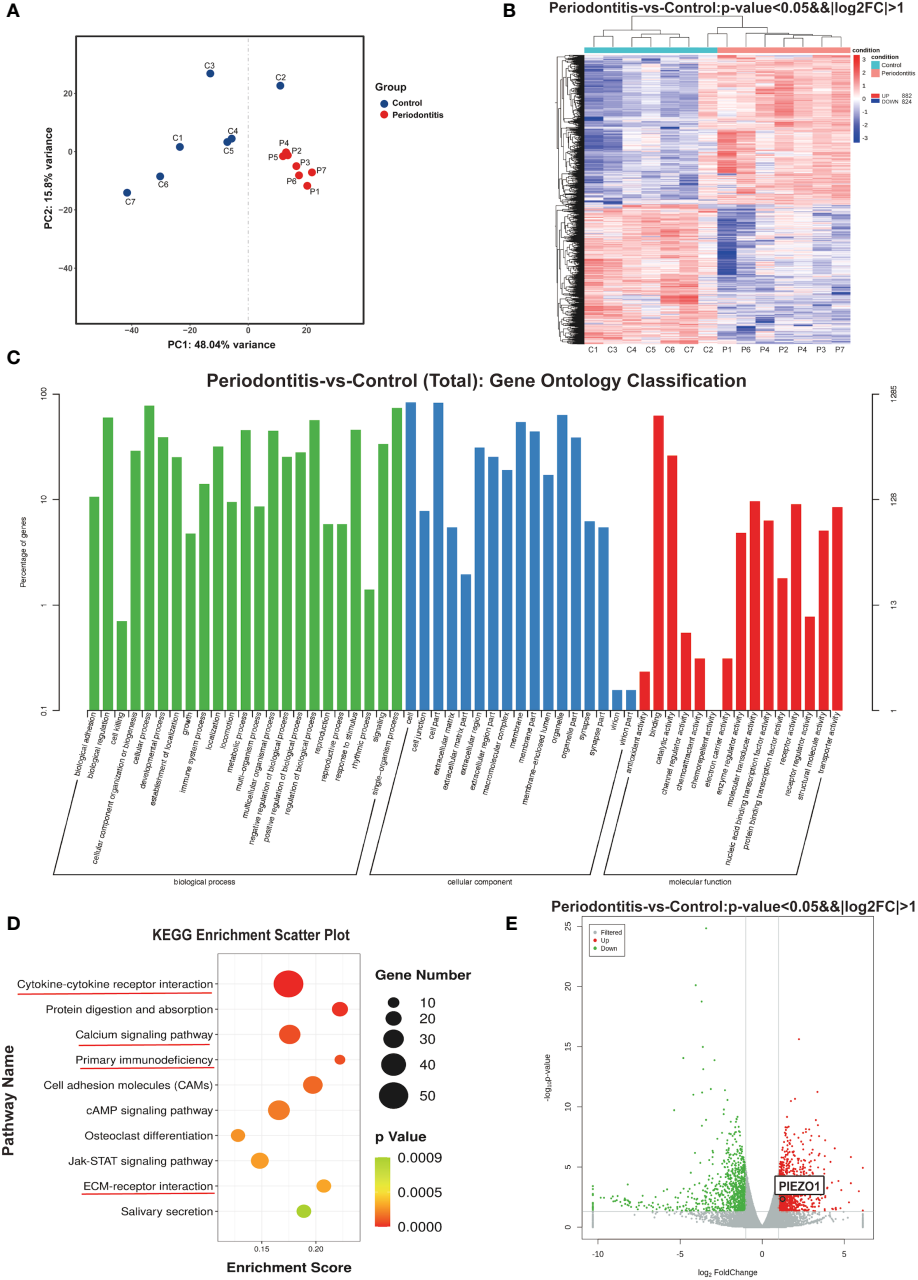
RNA sequencing results **(A)** PCA analysis examines the distribution of samples. **(B)** The heat map demonstrates that there are significant differences in gene expression between the two groups. **(C)** GO enrichment analysis results. **(D)** Bubble map of KEGG enrichment analysis of Top10 pathways. **(E)** Volcano plot showing genes differentially expressed between two groups. C, control group; P, periodontitis group; PCA, principal component analysis; GO, gene ontology; KEGG, Kyoto encyclopedia of genes and genomes.

Total RNA was isolated from seven samples of healthy gingival tissue and seven samples of gingival tissue affected by periodontitis as previously mentioned and cDNAs were then created from the RNA samples of both groups and sequenced by the Illumina HiSeq6000. The principal component analysis (PCA) results depicted the intergroup separation and aggregation tendencies among the control and periodontitis groups (**Figure 2A**). DESeq (18) was used to analyze gene expression differences between the two groups. A total of 1706 DEGs were detected after differential screening based on the expression of protein-coding genes in different samples. Among them, there were 882 up-regulated genes and 824 down-regulated genes (**Figure 2B**). The DEGs listed in **Supplementary Material 2** included inflammatory cytokines and immune response-related ones (*IL6*, *IL1RL1*, *IL17C*, *CSF3* and *CCL20*) and proteases (*MMP8*, *MMP13*, and *MME*) (**Figure S1**). Further GO analysis showed that these DEGs were mostly associated with “biological adhesion”, “cellular component organization or biogenesis” and “immune system process” in biological process (BP). They were also related to “extracellular matrix” in cellular component (CC) and “channel regulator activity” in molecular function (MF) (**Figure 2C**). Moreover, KEGG pathway analysis revealed that periodontitis mainly affects cytokine-cytokine receptor, primary immunodeficiency, calcium signaling pathway, and ECM-receptor interaction (**Figure 2D**). As a second messenger, calcium (Ca^2+^) is vital in the control of the immune response (19). Intriguingly, we found that Piezo1, a mechanosensitive ion channel, was significantly up-regulated in periodontitis group (**Figure 2E**). It was well documented that Piezo1 regulates the influx of Ca^2+^ into cells, thus converting mechanical signals into chemical signals (16). Recent studies showed that Piezo1 modulates inflammatory response, indicating Piezo1 might play an important role in immunity (20). Therefore, we chose the *PIEZO1* as the key gene for following research.

### 3.3 Piezo1 was upregulated in macrophages of periodontitis gingival tissues and induced by LPS

**Figure 3:**
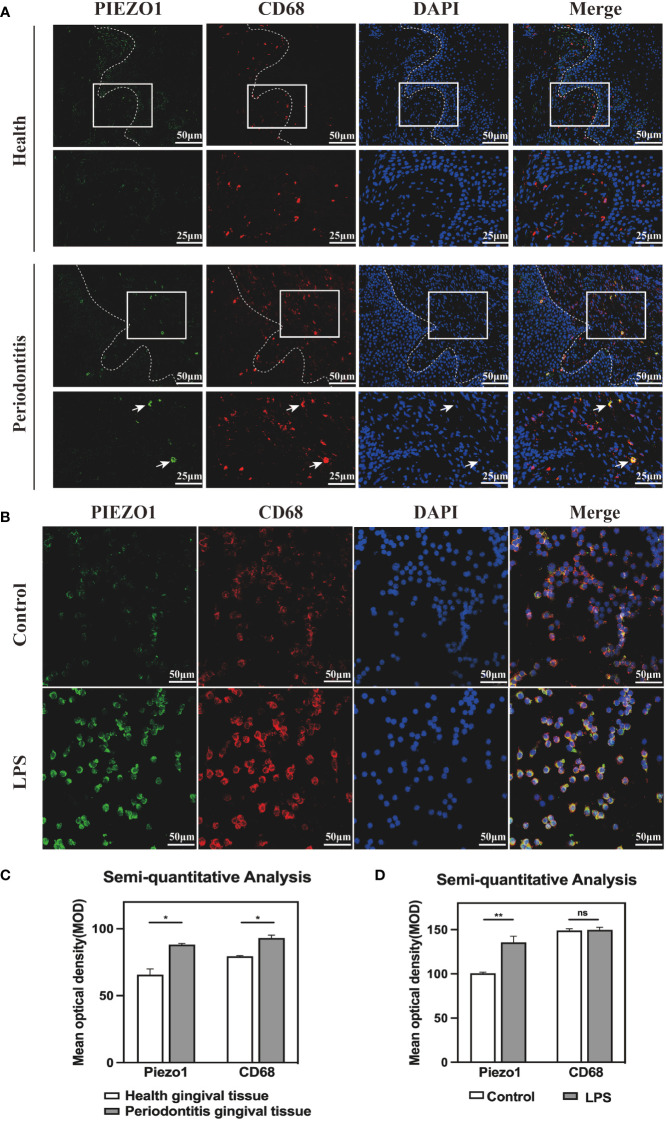
Piezo1 was located in macrophages **(A)** IF staining showed co-localization of PIEZO1 and CD68 in gingival tissue. **(B)** IF staining showed co-localization of PIEZO1 and CD68 in RAW264. 7. **(C)** Semi-quantitative analysis of PIEZO1 and CD68 in gingival tissue. **(D)** Semi-quantitative analysis of PIEZO1 and CD68 in RAW264. 7. *P < 0. 05; **P < 0.01; ns, non significance..

CD68 is a pan macrophage surface marker. To confirm whether Piezo1 existed in macrophages, we carried out IF staining in healthy and periodontitis gingival tissues, as well as in RAW264. 7 stimulated with LPS. IF results revealed Piezo1 located in macrophages (labeled by CD68) of periodontitis gingival tissues (**Figure 3A**) and in the LPS-stimulated RAW264. 7 (**Figure 3B**). Semi-quantitative analysis showed higher levels of Piezo1 in periodontal tissues than in healthy gingival tissues, and in LPS-stimulated RAW264. 7 cells than control cells (**Figures 3C, D** **)**. Taken together, Piezo1 is upregulated in macrophages of periodontitis gingival tissues and is induced by LPS.

### 3.4 Piezo1 blockade attenuated M1 macrophage polarization and pro-inflammatory cytokine production in response to LPS

M1 macrophage produce pro-inflammatory cytokines leading to periodontitis. To elucidate whether Piezo1 has an effect on M1 macrophage differentiation and cytokine production, we used GsMTx4, an inactivating non-selective cationic MSC inhibitor known to inhibit Piezo1 activity (21, 22). Although it was reported that GsMTx4 at a low dose (e. g. 0. 5μM) has no apparent effect on the cell viability of astrocytes (23), we determined if it has such an effect in macrophages at a high dose in the presence of LPS. Thus, Raw264. 7 cells were treated with GsMTx4 at 2. 5, 4, or 10μM for up to 4 days. We found that GsMTx4 at the doses up to 4μM had no apparent effect on the cell viability, whereas it at 10μM dramatically reduced the cell viability (**Figure S2A**). Therefore, GsMTx4 at 4μM was used as the treatment dose in the following experiments.

**Figure 4:**
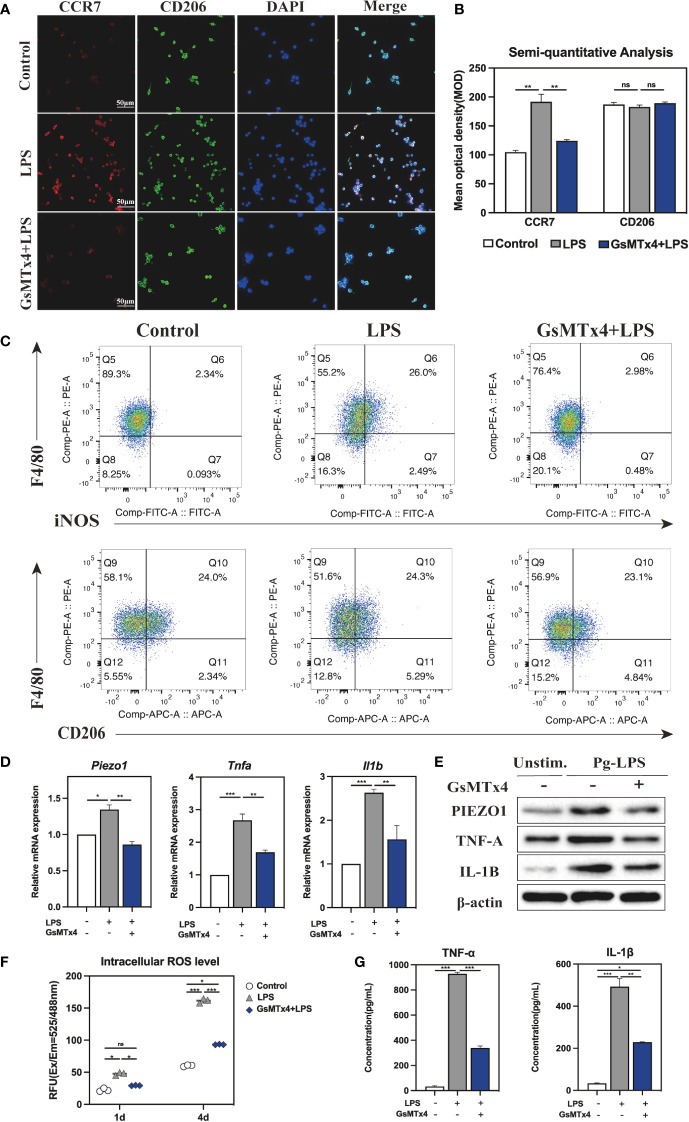
Immune response of macrophages after blocking Piezo1. **(A)** IF staining of CCR7 and CD206 in the Control, LPS and GsMTx4+LPS groups after 4-day-cultured. M1 macrophages were marked with CCR7 (red), M2 macrophages with CD206 (green), and nuclei with DAPI (blue). **(B)** Semi-quantitative analysis of CCR7 and CD163 in each group. **(C)** Flow cytometry analysis of RAW264. 7 cells in the Control, LPS and GsMTx4+LPS groups. Q6 represents M1 types (F4/80^+^/iNOS^+^) and Q10 represents M2 types (F4/80^+^/CD206^+^). **(D)** Expressions of *Piezo1* and inflammation-related genes (*Tnfa* and *Il1b*) in macrophages cultured for 4 days. **(E)** Western blotting analysis of PIEZO1, IL-1B and TNF-A in RAW264. 7 cultured for 4 days. **(F)** Intracellular ROS levels of RAW264. 7 cultured for 4 days. **(G)** Concentration of inflammatory cytokines in macrophage medium detected by ELISA. *P < 0. 05; **P < 0. 01; ***P < 0. 001.

Next, immunofluorescence (IF) staining was used to analyze macrophage polarization, which was closely related to the inflammatory response. Markers CCR7 and CD206 were respectively used to designate M1 (red color) and M2 (green color). The polarization of stained macrophages cultivated under different conditions was observed (**Figure 4A**). Compared with the Control and GsMTx4+LPS groups, the expression of CCR7 in the LPS group was noticeably higher, and however, CD206 did not differ significantly among the three groups. Semi-quantitative analysis showed that M2 macrophages had similar polarization ratio on each group, but the polarization trend of M1 macrophages in the LPS group was higher than that in the GsMTx4+LPS group and the Control group (**Figure 4B**). A further analysis was conducted by flow cytometry to determine the proportions of macrophage phenotypes. F4/80, iNOS and CD206 were selected as markers of macrophages, M1 phenotypes and M2 phenotypes, respectively. As shown in **Figure 4C**, Q6 represents M1 types (F480^+^/iNOS^+^), while Q10 represents M2 types (F480^+^/CD206^+^). The LPS group contained 26. 0% M1, which was much higher than the Control group (2. 34%) and GsMTx4+LPS group (2. 98%) (P<0. 05). Three groups had nearly accounted M2 phenotypes with a proportion of 21%, 24. 5%, and 24. 8%, respectively, which did not differ statistically (P>0. 05, **Figure S2B**). These results demonstrated that inhibition of Piezo1 affected macrophage polarization toward M1 in response to LPS stimulation.

The macrophage inflammatory response was evaluated by qRT-PCR, Western blotting, and intracellular ROS analysis. As for the expression of mRNA, LPS-stimulated macrophage considerably raised, whereas GsMTx4 significantly decreased the expression of *Piezo1* (P<0. 05) (**Figure 4D**). TNF-α and IL-1β are potent pro-inflammatory cytokines produced by activated M1 macrophages. The results showed that LPS dramatically enhanced the expression of *Tnfa* and *Il1b* in macrophages, which was markedly inhibited by GsMTx4. The same outcomes were attained for Western blotting analysis (**Figure 4E**, **Figure S2C**). Aside from secreting cytokines such as TNF-α and IL-1β, M1 macrophages produce ROS in significant quantities, which is essential to the antimicrobial response. The intracellular ROS analysis showed a significant low level of ROS in the GsMTx4+LPS group compared to the LPS group in 4-day-cultured RAW264. 7 cells (**Figure 4F**), suggesting that blocking Piezo1 reduced ROS production by LPS in macrophages.

For further illustration, ELISA was utilized to determine the cytokines levels in macrophage 4-day-cultured medium under various culture conditions. The results showed that GsMTx4 markedly inhibited LPS-induced production of TNF-α and IL-1β in RAW264. 7 cells (**Figure 4G**). Collectively, these data suggest that blocking Piezo1 attenuates macrophage polarization to M1 and decreases the production of inflammatory factors.

### 3.5 Piezo1 stimulation enhanced M1 polarization and production of pro-inflammatory cytokines in response to LPS

**Figure 5:**
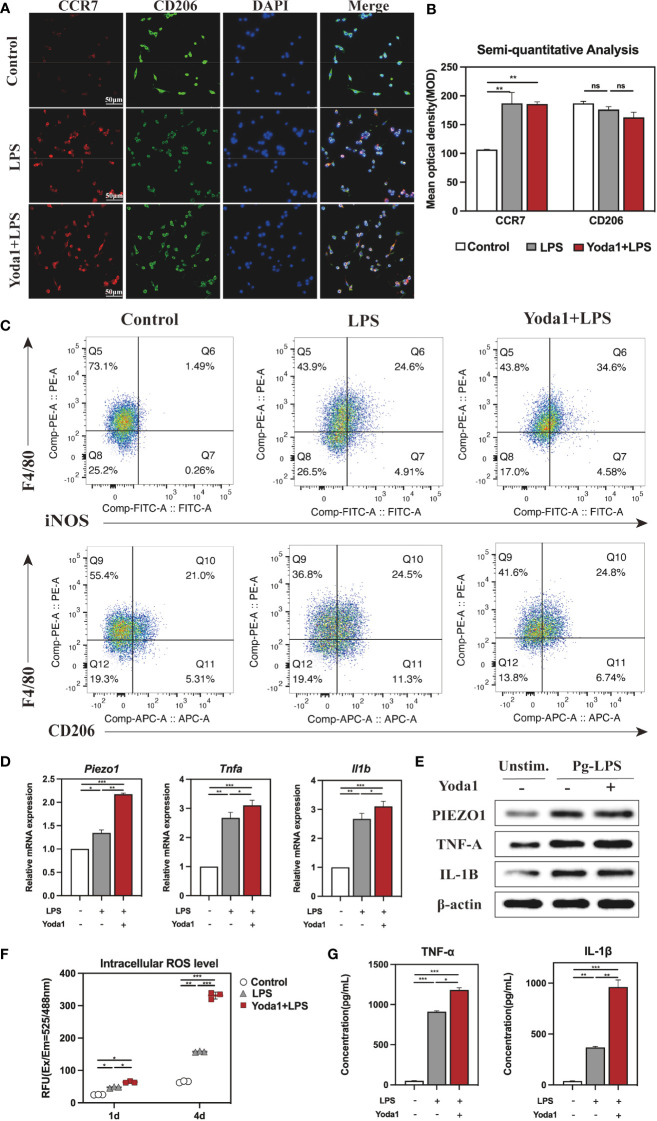
Immune response of macrophages after activating Piezo1. **(A)** IF staining of CCR7 and CD206 in the Control, LPS and Yoda1+LPS groups after 4-day-cultured. M1 macrophages were marked with CCR7 (red), M2 macrophages with CD206 (green), and nuclei with DAPI (blue). **(B)** Semi-quantitative analysis of CCR7 and CD206. **(C)** Flow cytometry analysis of RAW264. 7 cells in the Control, LPS and Yoda1+LPS groups. Q6 represents M1 types (F4/80^+^/iNOS^+^) and Q10 represents M2 types (F4/80^+^/CD206^+^). **(D)** *Piezo1*, *Tnfa* and *Il1b* genes expressions in macrophages cultured for 4 days. **(E)** Western blotting analysis of PIEZO1, TNF-A and IL-1B in RAW264. 7 cultured for 4 days. **(F)** Intracellular ROS levels of RAW264. 7 cultured for 4 days. **(G)** Concentration of inflammatory cytokines in macrophages medium detected by ELISA. *P < 0. 05; **P < 0. 01; ***P < 0. 001.

To further understand how Piezol triggers the immune response, we treated macrophages with Yoda1, a Piezo1 selective agonist (24). Similar to processing with GsMTx4, we treated RAW264. 7 with 2, 5, or 10μM of Yoda1 in the presence of LPS and found that Yoda1 had no apparent effect on the cell viability and eventually selected 5μM Yoda1 to stimulate the cells (**Figure S3A**). Similarly, CCR7 and CD206 were employed to mark M1 and M2 types, respectively, to analyze the polarization of macrophages. The IF staining results visually demonstrated that the red fluorescence intensity representing CCR7 in the LPS group and the Yoda1+LPS group was significantly stronger than that of the Control group, indicating that the first two groups included a higher proportion of M1 macrophages (**Figure 5A**). Semi-quantitative analysis demonstrated that the mean optical density of red was significantly higher in the LPS and the Yoda1+LPS groups than in the Control group (**Figure 5B**). Flow cytometry results (**Figure 5C**) showed that there were 34. 6% M1 types in the Yoda1+LPS group, 24. 6% in the LPS group, and only 1. 49% in the Control group. In contrast, the percentage of M2 types in each group did not differ (**Figure S3B**). These findings suggest that Piezo1 agonist can enhance LPS-induced M1 polarization but has no apparent effect on M2 polarization.

Moreover, qRT-PCR results revealed that the expressions of *Piezo1*, *Tnfa* and *Il1b* were significantly higher in groups of LPS and Yoda1+LPS than those in the Control group (**Figure 5D**), and Western blotting analysis yielded similar results (**Figure 5E**, **Figure S3C**). As expected, ROS levels in various groups for 4 days rose in the order of Control < LPS < Yoda1+LPS (**Figure 5F**). ELISA showed that the highest concentrations of TNF-α and IL-1β in macrophage supernatants were reported in the Yoda1+LPS group compared to the Control and LPS groups (**Figure 5G**). Taken together, boosting Piezo1 can excessively enhance macrophage M1 polarization and the production of inflammatory factors in response to LPS.

### 3.6 Macrophage-mediated MMPs induced collagen degradation via Piezo1

A major component of periodontal extracellular matrix is type I collagen (COL-I) and type III collagen (COL-III). Our RNA-seq results showed upregulated MMP8 and MMP13 in periodontitis group, which were reported to be involved in the degradation of COL-I, COL-III and extracellular matrix observed in periodontitis (25, 26). It is well known that immunological dysregulation lead to tissue damage (27). Therefore, we hypothesized that Piezo1 stimulated excessive immune response which in turn increased the expression of collagenase (such as MMP8 and MMP13) and led to collagen degradation. To test our hypothesis, we treated HGF-1 cells with the diluted macrophage-derived condition medium (MDCM), which was collected the LPS, the Yoda1+LPS, the GsMTx4+LPS, or the Control groups after cultured for 4 days. The MDCM was combined with fresh DMEM medium in a ratio of 1:3 to create conditioned medium for culturing HGF-1 cells, and each group was named L1D3, Y1D3, G1D3 and C1D3, respectively. The CCK8 assay showed that each MDCM had no effect on HGF-1 cell viability at 1 day. When HGF-1s were conditioned cultured for 4 and 7 days, the C1D3, L1D3, or G1D3 had no significant effect on HGF-1 cell viability, but the Y1D3 produced a mildly inhibitory effect on HGF-1s, suggesting that excessive inflammatory responses affect cell viability (**Figure S4**).

**Figure 6:**
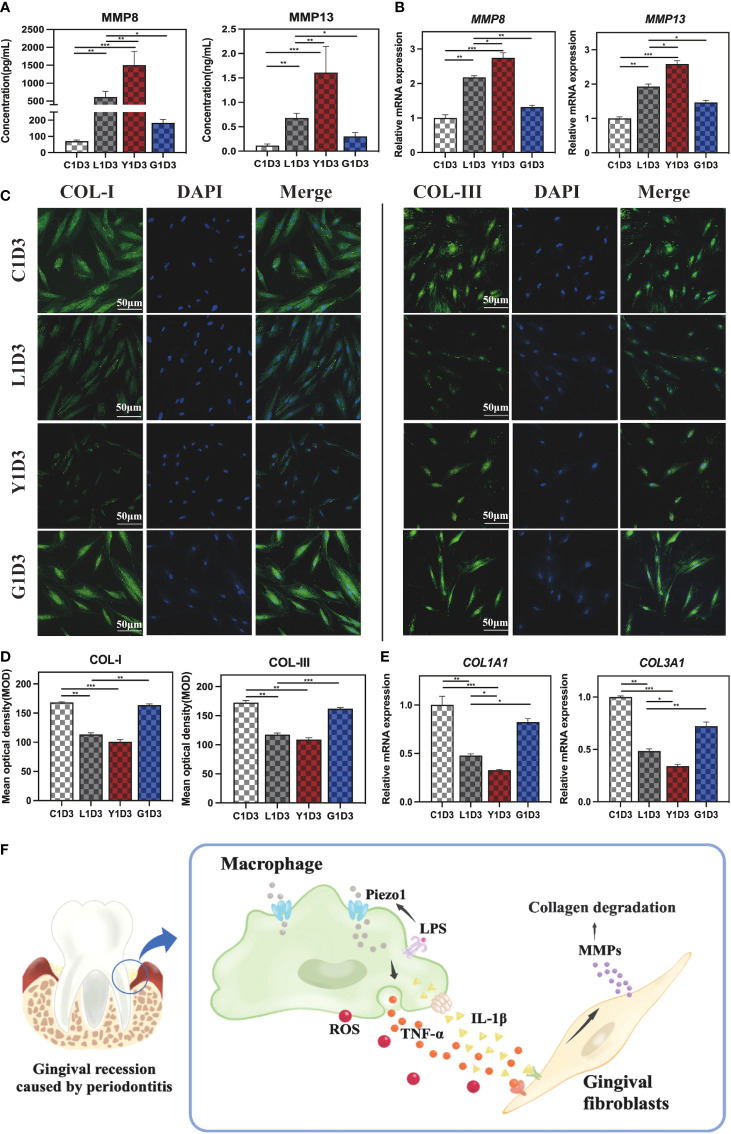
Gingival fibroblasts cultured in conditioned medium. **(A)** Concentration of MMP8 and MMP13 in the supernatant of HGF-1 in each group detected by ELISA. **(B)** *MMP8* and *MMP13* gene expressions in HGF-1 cultured in MDCM for 7 days. **(C)** IF staining of COL-I and COL-III in HGF-1 cultured with MDCM for 7days. **(D)** Semi-quantitative analysis of COL-I and COL-III in HGF-1. **(E)** Gene expression of *COL1A1* and *COL3A1* in HGF-1 cultured with MDCM for 7 days. **(F)** Schematic diagram of macrophages mediating MMPs-degrading collagens via Piezo1, thereby destroying periodontal tissue. *P < 0. 05; **P < 0. 01; ***P < 0. 001.

Macrophages interact strongly with other cells through paracrine secretion. ELISA was employed to measure collagenase MMPs concentration in order to verify whether MDCM alters MMPs secreted from HGF-1s. As shown in **Figure 6A**, the concentrations of MMP8 and MMP13 were increased in the L1D3 and Y1D3 groups compared to the C1D3 group (P<0. 01). Compared to the L1D3 group, the Y1D3 group secreted higher levels of MMP8 and MMP13 (P<0. 01), whereas the G1D3 group secreted less MMPs (P<0. 05), indicating that MDCM affects the secretion of MMPs from HGF-1. Moreover, qRT-PCR was used to assess MMPs expression level in HGF-1 cells. The results showed that MMP8 and MMP13 were upregulated in the L1D3 and Y1D3 groups, while considerably downregulated in the G1D3 group (P<0. 01, **Figure 6B**). The results suggest that LPS-stimulated macrophages or the activation of Piezo1 by Yoda1 both promoted downstream MMPs expression, whereas block of Piezo1 significantly inhibited the LPS-induced MMP expression. Taken together, macrophages affected the expression and secretion of MMPs in HGF-1 through Piezo1. Activating Piezo1 promoted the overexpression and secretion of collagenase MMPs, while inhibiting Piezo1 decreased the level of collagenase MMPs, suggesting that Piezo1 functioned as an upstream regulator of MMPs.

Furthermore, IF staining was used to assess the level of COL-I and COL-III in HGF-1s cultured with MDCM for 7 days. The results demonstrated that the trend of COL-I and COL-III was as follows:*C1D3*≈*G1D3*>*L1D3*≈*Y1D3* (**Figure 6C**). Semi-quantitative analysis showed the *L1D3* and *Y1D3* groups were much lower than that of the *C1D3* group for both COL-I and COL-III, while there was no obvious difference in the *G1D3* group (**Figure 6D**). Further validation by qRT-PCR revealed that the expression of *COL1A1* and *COL3A1* in the L1D3 and Y1D3 groups was markedly reduced, while the expression in the G1D3 group was not significantly altered compared with the C1D3 group (**Figure 6E**). These data suggest that activating Piezo1 in macrophages can exacerbate the downregulation of collagen-related gene and protein expression, thereby promoting collagen degradation, while blocking Piezo1 can counteract this damage.

## 4 Discussion

Accordingly, we discovered a novel mechanism underlying periodontitis-related gingival destruction mediated by Piezo1 in this research. Our results showed that Piezo1 is substantially expressed in periodontitis tissues, and it may operate on macrophages to modulate the immune response. We found that inhibition or activation of Piezo1 affects macrophage polarization in an inflammatory milieu and modulates MMPs’ secretion. We further revealed that macrophages mediate MMPs via Piezo1 to regulate collagen degradation in gingival fibroblasts. The schematic diagram of this research was displayed in **Figure 6F**. These results offered fresh perceptions into possible mechanisms underlying periodontitis-induced gingival destruction.

Periodontitis is a widely prevalent infectious periodontal disease and is characterized by the destruction of tooth-supporting tissues such as alveolar bone resorption and gingival destruction (28, 29). It is initiated by microbial infection in dental plaque and interacts with the host immune defense, while macrophages play a crucial role in activating the host immune defenses and defending against pathogenic bacterial infections (30). On one hand, macrophages migrate and aggregate to the site of infection, acting as phagocytic bactericides. On the other hand, macrophages secrete numerous cytokines and amplify specific immune responses upon contact with microorganisms (31), thereby inducing inflammation and stimulating an increase in the destruction of collagen fibers in the gingiva, and ultimately leading to gingival atrophy (32). However, the mechanism underlying this double-edged role of macrophages in periodontal inflammation remains elusive. Ca^2+^ concentration affects the inflammatory response by polarizing macrophages toward M1 (33, 34). The nonselective ion channel Piezo1 has been demonstrated to regulate Ca^2+^ influx into macrophages (10, 35), thereby responding to macrophage activation and eliciting an inflammatory response (16, 20, 36). Toll-like receptor 4 (TLR4) and Piezo1 collaborate to mediate Ca^2+^ influx in response to LPS stimulation, enhancing macrophage activity (14). But the role of Piezo1 in modulating periodontal inflammation is undetermined. To bridge this knowledge gap, we determined that Piezo1 functions on macrophages in periodontal inflammatory tissues by IF staining co-localization techniques on infected gingival tissue and macrophages stimulated by Pg-LPS. In periodontitis, activated Piezo1 causes a large extracellular Ca^2+^ influx into macrophages, leading to an increase in M1-type polarization. Then excessive M1 macrophages cause exaggerated inflammatory responses by overproduction of ROS and pro-inflammatory factors (such as TNF-α and IL-1β), in turn resulting in irreversible destruction of periodontal tissues.

It is widely accepted that the component analysis of gingival crevicular fluid (GCF) can shed light on the association between particular metabolic alterations and periodontitis states (25, 37, 38). MMP8 and MMP13 are generally acknowledged to be the most rewarding salivary biomarkers for the diagnosis of periodontitis (25, 39), which are crucial proteases that regulate gingival destruction and alveolar bone destruction in periodontitis (40). Similarly, our transcriptome analysis results from gingival tissue showed that the periodontitis group had considerably greater expression of multiple MMPs, including collagenases (MMP8 and MMP13), than the healthy group, supporting the opinion that these MMPs can be employed as periodontitis biomarkers. An overabundance of MMPs can induce periodontal tissue destruction, fibrosis, and degradation of the extracellular matrix, ultimately leading to loss of connective tissue attachment and gingival destruction (41). Macrophage-derived pro-inflammatory factors are associated with tissue damage in periodontitis, and among them MMP is one of the most strongly associated and extensively researched protease families (42). Our data suggested that macrophages can regulate the secretion of MMP8 and MMP13 *via* activating Piezo1, which in turn affects the collagen level of gingival fibroblasts through the cytokines-rich conditioned medium. However, how the protein hydrolytic activity of intracellular MMPs is regulated remains unclear. Most currently believe that ROS as a signaling molecule may be a possible mechanism to regulate intracellular MMPs activity (43, 44). Oxidative stress can enhance MMP activation and is associated with MMP function. Jing Geng et al. confirmed that Piezo1 can enhance macrophage activity and ROS accumulation (14). Our study yielded the same results. We hold the view that hyperactivated M1 macrophages generate more ROS via Piezo1, causing oxidative stress and hence enhancing MMPs secretion and consequently leading to collagen breakdown. After all, the fundamental mechanism of macrophage control of Piezo1-mediated MMPs needs further investigation.

Using macrophage conditioned medium to culture HGFs for the purpose of evaluating the impact of the former’s paracrine factors on the latter can effectively imitate the complicated milieu of periodontitis *in vitro* (45). In this manuscript, collagen expression in HGF was influenced by Piezo1-mediated MMPs through the macrophage conditioned medium. Logically, the degree of collagen degradation is remarkably consistent with the concentration of MMPs. In an inflammatory context, Piezo1 enhancement further diminished fibroblast collagen expression, whereas Piezo1 inhibition noticeably counteracted inflammation-induced collagen degradation.

To summarize, we proposed a novel mechanism underlying periodontitis-induced gingival destruction: macrophages mediate MMPs-degrading collagens via Piezo1, thereby destroying periodontal tissue. Cellular crosstalk between macrophages and fibroblasts plays an essential role in periodontitis pathogenesis. Our findings provide fresh insights into this complicated molecular mechanism, possibly providing a new treatment strategy for periodontitis-induced soft tissue atrophy. Nevertheless, there is still a long way to go before we fully elucidate the biological function and specific mechanism of how Piezo1 mediate MMPs and affects the periodontitis-induced gingival destruction.

## Data availability statement

The data presented in the study are deposited in the NCBI Sequence Read Archive (SRA) repository, accession number PRJNA967820 (https://www.ncbi.nlm.nih.gov/sra/PRJNA967820).

## Ethics statement

The studies involving human participants were reviewed and approved by Medical Ethics Committee of the School of Stomatology, Nanjing Medical University and the Affiliated Stomatology Hospital of Nanjing Medical University. The patients/participants provided their written informed consent to participate in this study.

## Author contributions

TZ and CT designed the study. ZC and CC collected and analyzed the data. TZ finished the experiments. SD sourced the literature. GL and JW edited the manuscript. CT provided the funding and supervised the whole study. All authors contributed to the article and approved the submitted version.
