# Supplementary material for: Macrophages induce gingival destruction via Piezo1-mediated MMPs-degrading collagens in periodontitis
Source: Front Immunol. 2023 May 16;14:1194662. doi: 10.3389/fimmu.2023.1194662 (PMC10228731; doi:10.3389/fimmu.2023.1194662)
Supplement: Supplementary file 1 [file DataSheet_1.docx]

Supplementary Material

Macrophages induce periodontitis-related gingival recession via Piezo1-mediated MMPs-degrading collagens

Tong Zhao, Zhuangzhuang Chu, Catherine Huihan Chu, Shuo Dong Guoqing Li, Jin Wu, Chunbo Tang*

*** Correspondence:** Chunbo Tang: cbtang@njmu.edu.cn

# Supplementary Figures and Tables

## Supplementary Figures


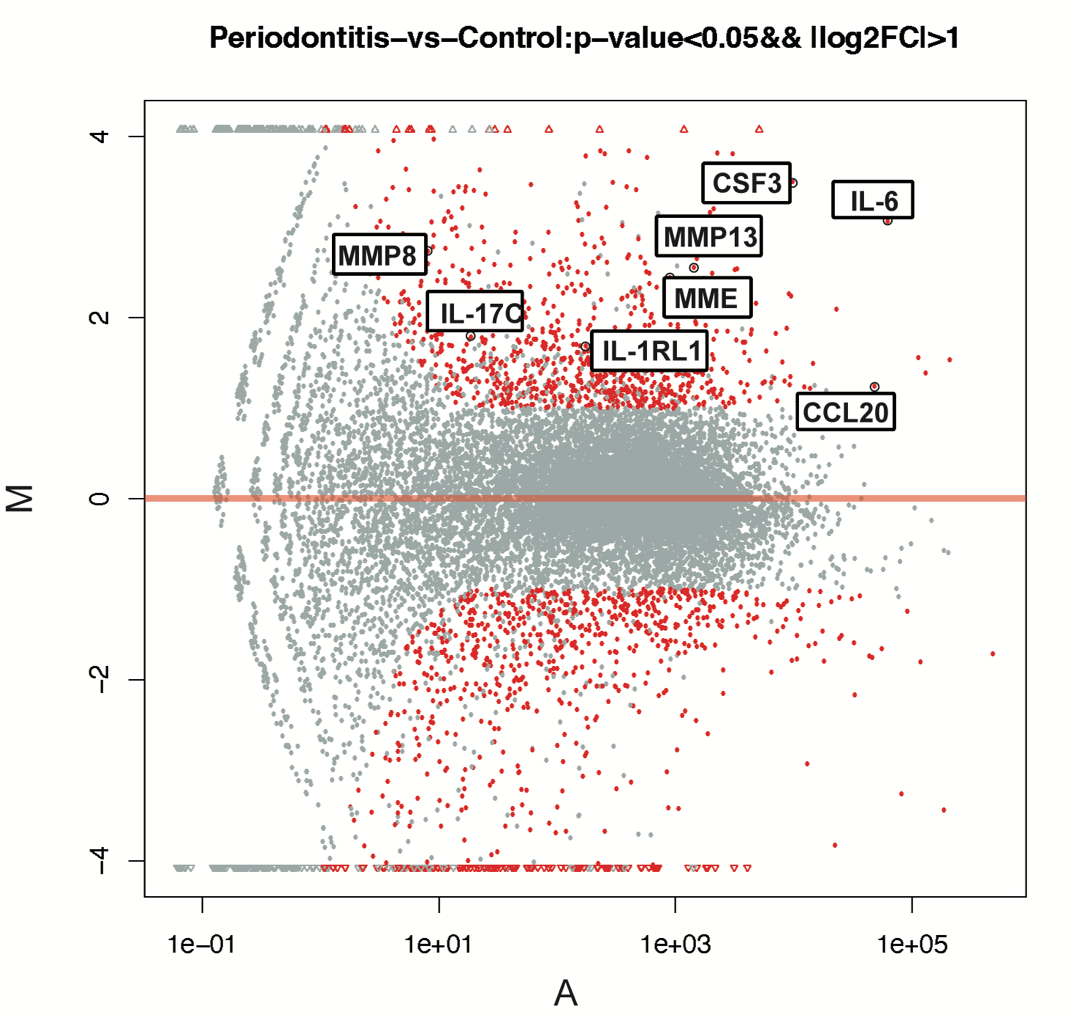


**Supplementary Figure 1.** The MA map demonstrated the difference in gene expression of *IL-6*, *IL-1RL1*, *IL-17C*, *CSF3*, *CCL20*, *MMP8*, *MMP13* and *MME* between periodontitis and control groups. C: control group, P: periodontitis group.


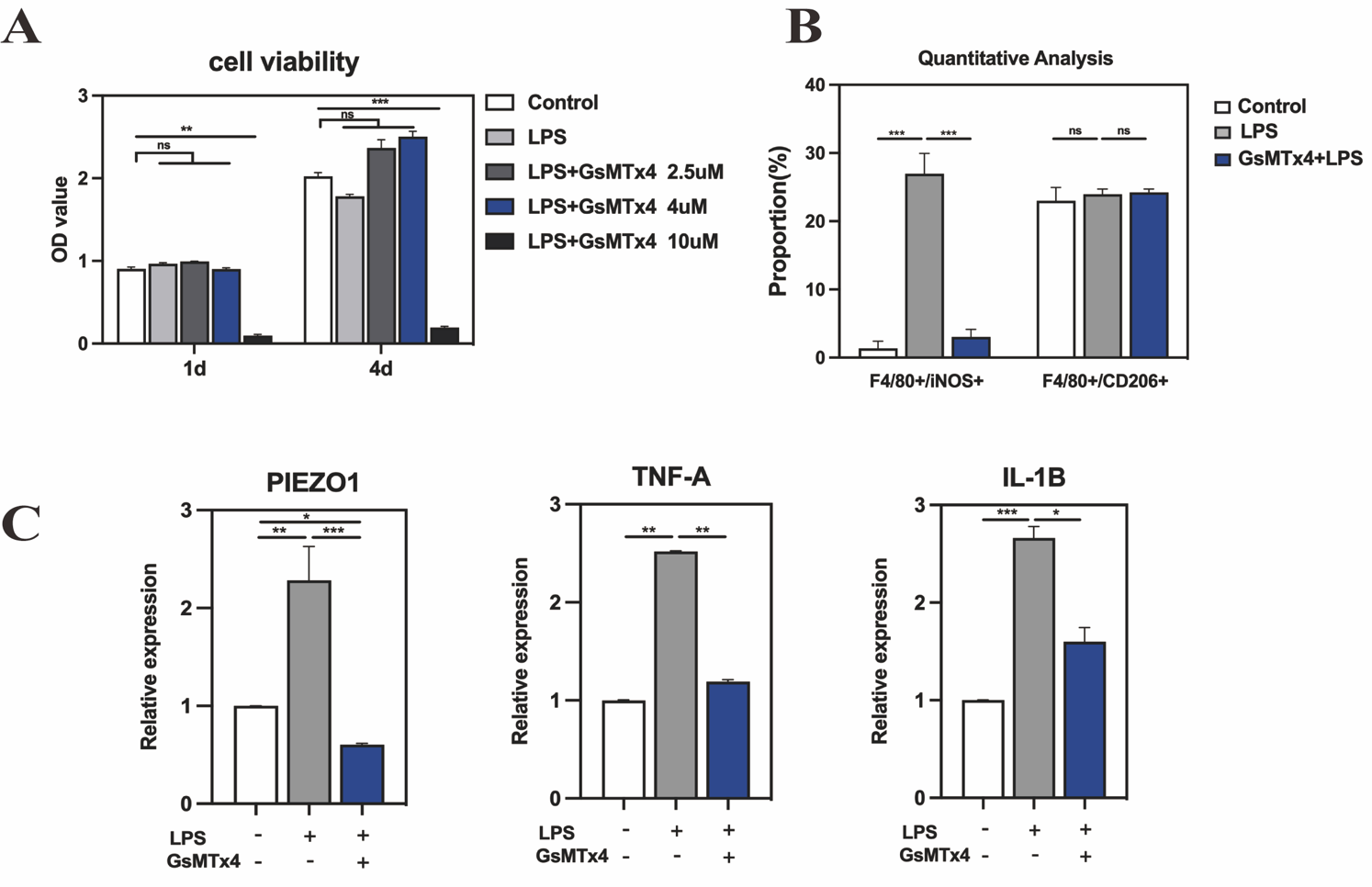


**Supplementary Figure 2.** Supplementary data of RAW264.7 treated with GsMTx4. **(A)** RAW264.7 viability treated with different concentrations of GsMTx4. **(B)** Quantitative analysis of F4/80^+^/iNOS^+^ and F4/80^+^/CD206^+^ in RAW264.7 in Control, LPS and GsMTx4+LPS group. **(C)** Semi-quantitative analysis of PIEZO1, TNF-A and IL-1B in RAW264.7 in Control, LPS and GsMTx4+LPS group. *P< 0.05; **P < 0.01; ***P< 0.001.


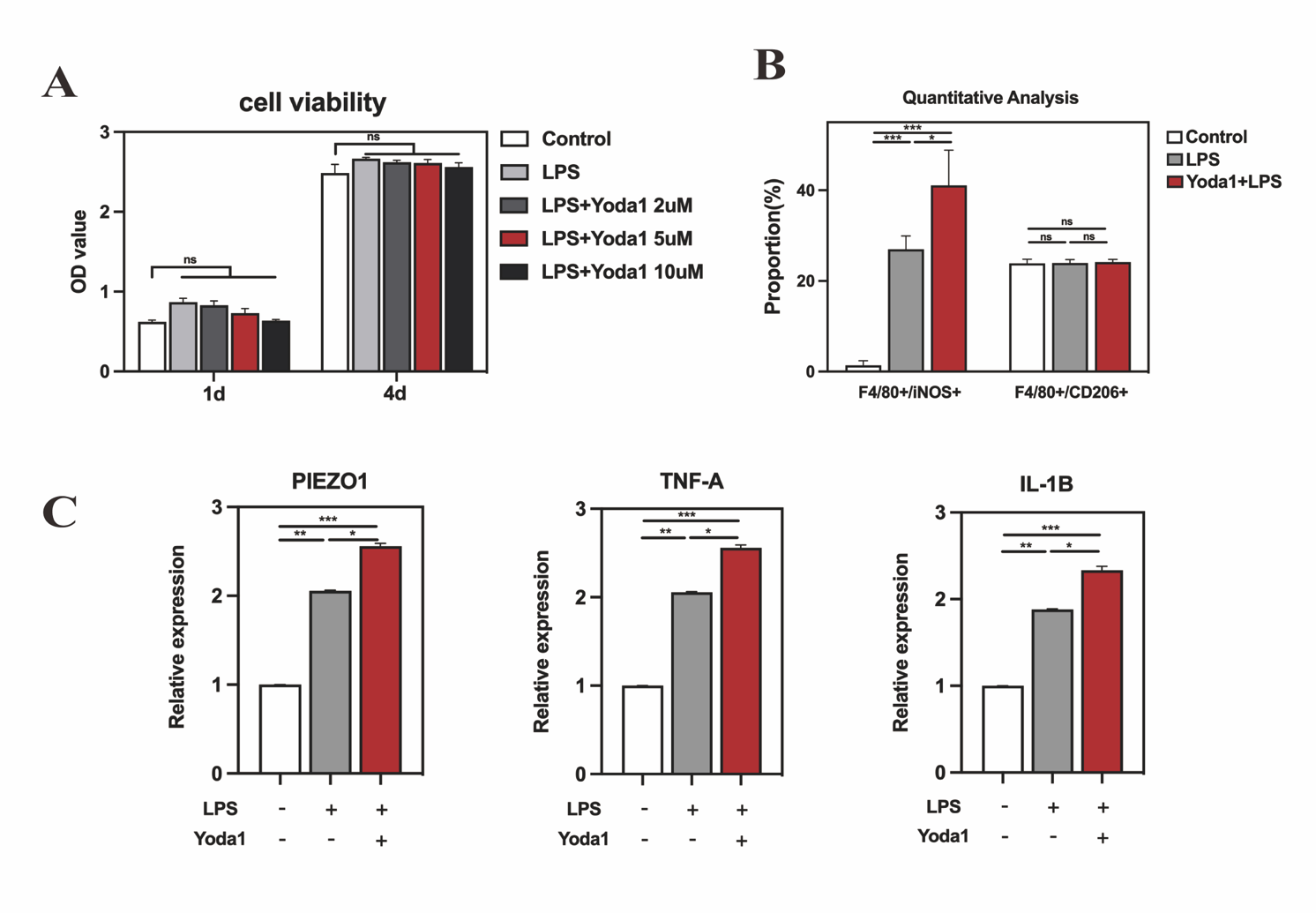


**Supplementary Figure 3.** Supplementary datas of RAW264.7 treated with Yoda1. **(A)** RAW264.7 viability treated with different concentrations of Yoda1. **(B)** Quantitative analysis of F4/80^+^/iNOS^+^ and F4/80^+^/CD206^+^ in RAW264.7 in Control, LPS and Yoda1+LPS group. **(C)** Semi-quantitative analysis of PIEZO1, TNF-A and IL-1B in RAW264.7 in Control, LPS and Yoda1+LPS group. *P< 0.05; **P < 0.01; ***P< 0.001.


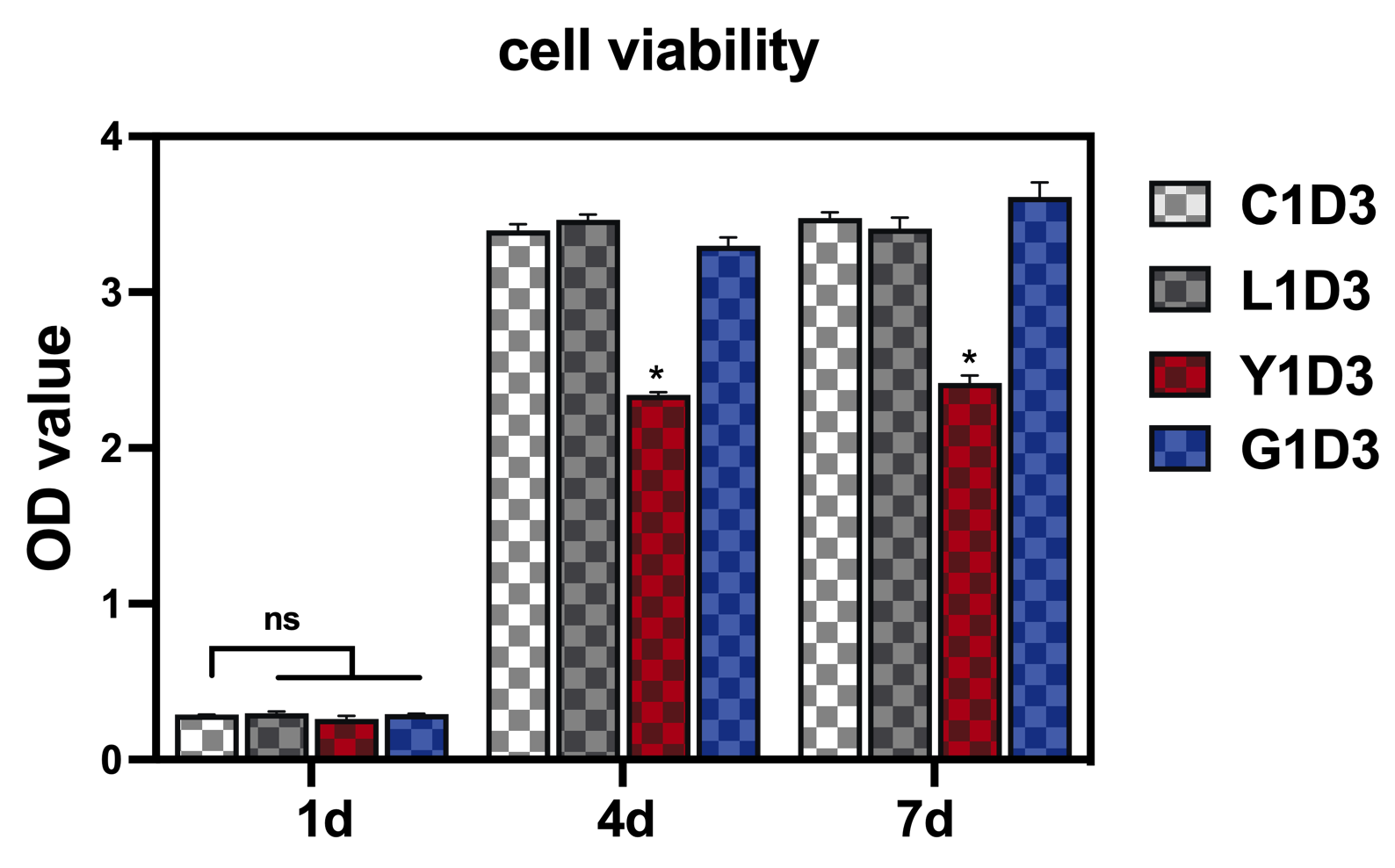


**Supplementary Figure 4.** HGF-1 cell viability treated with RAW264.7 conditioned medium.

## Supplementary Tables

**Supplementary Table 1**. Primers used for qRT-PCR in the immunological evaluation.

| Gene (mouse) | Primer sequences  (F, forward; R, reverse; 5’-3’) | Product size (bp) |
| --- | --- | --- |
| *Actb* | F: CCTCTATGCCAACACAGT  R: AGCCACCAATCCACACAG | 155 |
| *Tnfa* | F: TTAGAGCGGGATAGTAACG  R: CAAAATACACAACAGTGTC | 111 |
| *Il1b* | F: TCCAGGATGAGGACATGAGCAC  R: GAACGTCACACACCAGCAGGTTA | 135 |
| *Piezo1* | F: GCTATCCCAAGGACCATTGC  R: CTGTCTCAGACTGCGATTCCA | 90 |

**Supplementary Table 2**. Primers used for qRT-PCR in HGF-1

| Gene (human) | Primer sequences  (F, forward; R, reverse; 5’-3’) | Product size (bp) |
| --- | --- | --- |
| *ACTB* | F: CTCCATCCTGGCCTCGCTGT  R: GCTGTCACCTTCACCGTTCC | 268 |
| *COL1A1* | F: TAGTCTGTCCTGCGTCCTCTG  R: GAG TCT TTT GCT TCC TCC CAC | 97 |
| *COL3A1* | F: CTTCTCTTCCAGCCGAGCTTC  R: TGTGTTTCGTGCAACCATCC | 188 |
| *MMP8* | F: GAAGACAGAGGCAGAGATTGG  R: CAGGGTCACACTTGAAGGCTA | 127 |
| *MMP13* | F: GCAGTCTTTCTTCGGCTTAGA  R: TTGTATTCACCCACATCAGGA | 102 |

**Supplementary Table 3.** The characteristics of patients included in the study.

| Sample # | Healthy/Periodontitis | Gender | Age | PD  (mm) | CAL  (mm) | Gingival recession grade | BOP site |
| --- | --- | --- | --- | --- | --- | --- | --- |
| C1 | Healthy | M | 41 | 2.5 | 0 | - | 0 |
| C2 | Healthy | F | 33 | 2 | 0 | - | 0 |
| C3 | Healthy | M | 42 | 3 | 0 | - | 1 |
| C4 | Healthy | F | 45 | 3 | 0 | - | 0 |
| C5 | Healthy | F | 39 | 2 | 0 | - | 0 |
| C6 | Healthy | F | 38 | 2 | 0 | - | 0 |
| C7 | Healthy | M | 39 | 2.5 | 0 | - | 0 |
| P1 | Periodontitis | M | 53 | 7 | 9 | III | 2 |
| P2 | Periodontitis | M | 47 | 5 | 7 | II | 1 |
| P3 | Periodontitis | F | 52 | 6 | 7 | III | 1 |
| P4 | Periodontitis | M | 39 | 4.5 | 5 | II | 3 |
| P5 | Periodontitis | M | 42 | 5.5 | 7 | II | 1 |
| P6 | Periodontitis | F | 43 | 5 | 8 | III | 1 |
| P7 | Periodontitis | F | 51 | 6 | 7 | III | 2 |
